# Addressing Depression Symptoms among University Students under COVID-19 Restrictions—The Mediating Role of Stress and the Moderating Role of Resilience

**DOI:** 10.3390/ijerph182312752

**Published:** 2021-12-03

**Authors:** Chang Liu, Melinda McCabe, Sebastian Kellett-Renzella, Shruthi Shankar, Nardin Gerges, Kim Cornish

**Affiliations:** Turner Institute for Brain and Mental Health, School of Psychological Sciences, Monash University, Clayton, VIC 3800, Australia; chang.liu5@monash.edu (C.L.); Melinda.McCabe@monash.edu (M.M.); skel0011@student.monash.edu (S.K.-R.); Shruthi.Shankar@monash.edu (S.S.); Nardin.Gerges@monash.edu (N.G.)

**Keywords:** isolation, stress, resilience, depression symptoms, university students

## Abstract

Background: The COVID-19 pandemic has contributed to a decline in mental health globally. Compared to the general population, university students have been identified as a group vulnerable to developing depression symptoms during the pandemic. Social isolation, a signature mental health consequence under physical-distancing regulations, is a known predictor of depression symptoms during the pandemic. Yet, more research is required to understand the mechanism that underpins the isolation–depression association and identify psychological factors that may attenuate the association. The current study aimed to understand the role of stress and resilience in the isolation–depression association among university students. Methods: Data were collected from 1718 university students between 28 and 31 May 2020. Partial least squares structural equation modelling (PLS-SEM) was used to examine the mediating role of perceived stress and the moderating role of resilience in the isolation–depression association. Results: We found that perceived stress partially mediated the association between social isolation and depression symptoms. Both the direct and indirect effects were moderated by participants’ resilience levels. Conclusions: Social isolation during the pandemic may contribute to depression symptoms both directly and through elevated stress levels. As an internal strength, resilience may buffer the adverse effects of isolation and stress on depression symptoms. Targeted interventions including mindfulness and physical exercise training may provide promising results in reducing depression symptoms among university students and should be considered by university administrators particularly during times of imposed physical-distancing measures.

## 1. Introduction

The COVID-19 pandemic has caused public health concerns globally. Movement restrictions, including stay-at-home orders and physical-distancing rules, have been taken in most countries to some degree to reduce the spread of COVID-19. Despite its advantages in flattening the curve, the restriction regulations may bring up unfavourable mental health outcomes, including symptoms of depression. Research showed that the prevalence of moderately severe and severe depression symptoms among the general population (i.e., US adults) was 3.7-fold higher and 7.5-fold higher during the lockdown period than pre-pandemic, respectively [1].

University students may be at higher risk for experiencing depression symptoms during the pandemic. Compared to the general population, university students often face additional stressors, including changes in learning mode, uncertainty about academic trajectory and living arrangements, as well as a faltering job market after graduation. Previous research found that university students reported the highest prevalence of depression symptoms compared to medical staff and the general population; 38.6% vs. 21.2% vs. 15.8%, respectively [2]. Negative consequences and comorbidity associated with depression symptoms include substance misuse, insomnia and suicidal behaviours [3,4,5]. Given the high prevalence of depression symptoms among university students, it is important to understand risk/protective factors of depression symptoms and how they may be associated with depression symptoms during the pandemic. Such knowledge can help university administrators and mental health professionals develop targeted interventions to prevent the onset and progression of depression symptoms among students during the pandemic and pandemic recovery.

Social isolation (i.e., the feeling of disconnectedness with others and loneliness, [6]) has been quoted as ‘a signature mental health concern in the era of COVID-19’ and associated with depression symptoms [7]. The negative impact of social isolation on depression symptoms may be intensified during the pandemic due to publicly mandated restriction measures. Specifically, individuals were forced to withdraw from face-to-face interactions and social activities, resulting in shrunken social networks and increased feelings of isolation compared to pre-pandemic [8]. Despite examining the direct effect of isolation on internalized symptoms (e.g., depression symptoms), previous research highlighted the need to understand the mechanisms that underpin the isolation–depression association and identify psychological factors that may attenuate the association [9].

One construct that may explain the association between social isolation and depression symptoms is perceived stress (i.e., the degree to which individuals perceive life events as unpredictable, uncontrollable, and overwhelming [10]). Specifically, when under the same stressful situation, individuals in the top quintile of isolation scores tend to evaluate the situation as more intolerable and stressful than those in the bottom quintile [11]. The elevated stress level may further translate into depression symptoms as a stress reaction [12]. This aligns with past studies, which found stress was positively associated with depression symptoms [13,14,15,16]. Even pre-pandemic surveys from three European countries (i.e., Germany, Poland and Bulgaria) showed that perceived stress was strongly associated with depression symptoms among university students [13]. Further, perceived stress was identified as the strongest predictor of changes in depression symptoms during the pandemic across all age groups among the general population [17].

The indirect relationship between isolation, stress and depression may be due to differences in coping. Isolated individuals have been found to use less adaptive coping strategies (i.e., rumination) compared to their non-isolated counterparts. Additionally, the stress-buffer model suggests that individuals may be able to harness support from their social networks when facing stressful situations [18]. These strategies may help individuals appraise these situations as less severe and further reduce the risk of developing depression symptoms.

As previously noted, not all individuals experience the adverse mental health outcomes associated with social isolation. This inconsistency may suggest potential moderators that buffer the negative impact from isolation. One candidature moderator is resilience. Resilience is defined as the ability to bounce back from adversity and adapt to the changing environmental demands flexibly [19]. Resilience has been found as a positive predictor of psychological well-being (i.e., positive and sustainable mental state that enables individuals to function efficiently [20]), and negatively associated with depressive symptoms [21]. When facing internal and external stressors, resilient individuals may adjust themselves with positive re-evaluation and problem-solving strategies, reducing the negative emotions associated with stressful situations and overall stress levels [21,22]. For isolated individuals with limited access to coping resources from external social networks, resilience may serve as an internal strength, helping individuals adapt to adverse situations.

Taken together, the current study examined the mechanism linking isolation, stress and depression symptoms; we further explored whether the association may be moderated by resilience. Through a structural equation model, we proposed that (1) the relationship between social isolation and depression symptoms may be mediated by perceived stress, and (2) resilience may moderate the negative impacts of social isolation and stress on depression symptoms.

## 2. Materials and Methods

### 2.1. Participants and Procedure

Data were collected between 28 and 31 May 2020. All students who were currently enrolled with Monash University received invitation links to the online Qualtrics survey. Inclusion criteria for the current study are: (1) currently enrolled with Monash University; (2) aged 18 and above. Participants who did not provide informed consent were excluded from the current study. Only participants who completed all measures of interest were included in the data analysis, resulting in a final sample of 1718 participants. No reimbursement was provided for participation. This study followed the Helsinki Declaration and was approved by the Human Research Ethics Committee at Monash University (Project Number 23969).

### 2.2. Measures

Brief Resilience Scale [23]: This is a 6-item scale measuring resilience (i.e., the ability to bounce back under stressful situations; Smith et al., 2008). Sample items include ‘I tend to bounce back quickly after hard times’ and ‘It does not take me long to recover from a stressful event’. Responses range from 1 (Strongly Disagree) to 5 (Strongly Agree). The total score was the measure of interest for our study. The scale has been validated cross-culturally among different populations, with consistent reliability (Cronbach’s alpha ranging from 0.80 to 0.90, [24]). In the current study, the scale demonstrated good reliability (Cronbach’s alpha = 0.87). The overall average resilience score among clinical and non-clinical population is 3.7 (equivalent to 22.2 in total score prior to averaging [25]).

Patient-Reported Outcomes Measurement Information System (PROMIS) Depression Scale [26]: This is an 8-item scale measuring depressive symptoms in the past seven days. Sample items include ‘I felt depressed’ and ‘I felt like a failure’. Responses range from 1 (Never) to 5 (Always). The T-score was the measure of interest for our study. A cut-off score of 60 was used when screening for clinical depression [27]. The PROMIS Depression scale has been validated in both clinical and general populations, with consistent reliability (Cronbach’s alpha ranging from 0.95 to 0.97, [27,28,29]). In the current study, the scale demonstrated excellent reliability (Cronbach’s alpha = 0.95). The overall average resilience score among the general population is 50 [26].

Patient-Reported Outcomes Measurement Information System (PROMIS) Social Isolation Scale [26]: This is a 4-item scale measuring social isolation. Sample items include ‘I feel left out’ and ‘I feel isolated from others’. Responses range from 1 (never) to 5 (always). The T-score was the measure of interest for our study. The PROMIS social isolation scale has been validated among young adults, with excellent reliability (Cronbach’s alpha = 0.92 [6]). In the current study, the scale demonstrated good reliability (Cronbach’s alpha = 0.89). The overall average resilience score among the general population is 50 [26].

Perceived Stress Scale 10 items [30]: This is a widely used scale measuring perceived stress over the past month. Sample items include ‘In the last month, how often have you felt that you were unable to control the important things in your life’ and ‘In the last month, how often have you felt difficulties were piling up so high that you could not overcome them?’. Responses range from 0 (never) to 4 (very often). The total score was the measure of interest for our study. The PSS10 scale has been validated cross-culturally in both clinical and general populations, with consistent reliability (Cronbach’s alpha ranging from 0.74 to 0.91, [31]). In the current study, the scale demonstrated good reliability (Cronbach’s alpha = 0.89). The overall average score among the general population is 15.2 [32].

### 2.3. Analyses

This study aimed to examine the mediating role of perceived stress between social isolation and depressive symptoms among university students and whether resilience may moderate the effect of social isolation and perceived stress on depressive symptoms. The proposed model was examined via PLS-SEM. PLS-SEM is the second generation technique to analyse the causal relationship between latent variables [33]. Compared to the first generation technique (i.e., factor-based SEM analyses), the composite-based method (e.g., PLS-SEM) can ‘not only account for measurement error and consider the entire model structure in the parameter estimation, but also offer more flexibility in terms of model specification compared with the factor-based SEM methods’ [34]. These advantages make the PLS-SEM approach appropriate for the aim of the current study. The analysis procedures were conducted via the SmartPLS 3 software (SmartPLS GmbH, Bönningstedt, Germany). 

## 3. Results

Among 1718 participants, 1227 were females (71.4%). The median age of participants was 21 (SD = 8.02, aged 18–78). In total, 736 (42.8%) participants exhibited clinical levels of depression symptoms (T > 60). Descriptive characteristics and correlations of included variables were presented in Table 1. 

**Figure 1:**
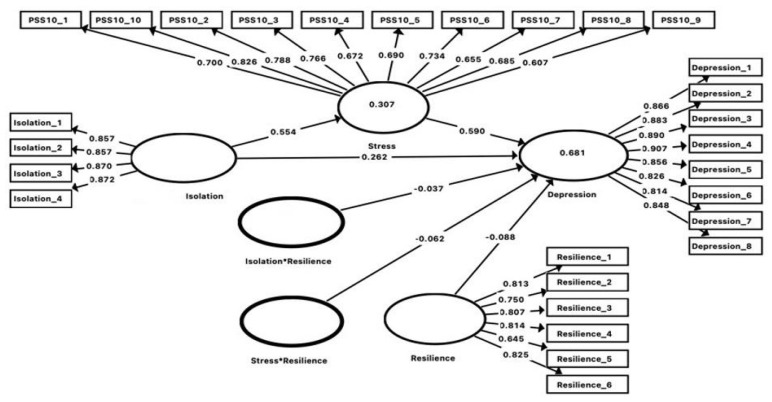
Conceptual model and PLS-SEM results.

The results of PLS-SEM analysis are presented in Figure 1, including showing the path coefficients (β) and the variance explained by the structural model (i.e., R^2^ values). As a prediction oriented approach, R^2^ was commonly used as an indicator of model fit for PLS-SEM. Based on [35], R^2^ values of 0.19, 0.33, and 0.67 refer to weak, moderate, and substantial variance explained by endogenous latent variables, respectively. For the current model, social isolation, stress and resilience accounted for 68.1% of the variance of depression symptoms. Meanwhile, social isolation accounted for 30.7% of the variance of perceived stress. 

To assess the reliability and validity of the measurement model, we calculated the internal consistency/reliability (indexed by Cronbach’s Alpha; Dijkstra–Henseler’s rho (pA) and composite reliability; see Table 2), convergent validity (indexed by the average variance extracted (AVE), see Table 2), and discriminant validity (the heterotrait–monotrait ratio (HTMT), see Table 3) for the measurement scales [36,37,38]. The measurement scales we used for the current study demonstrated good to excellent reliability (Cronbach’s alpha ≥ 0.80). All measurements meet the cut-off recommendation for composite reliability and rho_A ≥ 0.70 [38], indicating sufficient composite reliability. All measurement constructs met the recommendation for convergent validity (AVE ≥ 0.50 [38]). 

The HTMT values for measurement constructs were below 0.85 (see Table 3), indicating adequate discriminant validity [39].

To examine the mediation and moderation hypothesis, we used a bias-corrected bootstrap procedure to determine the statistical significance of coefficients [34]. Based on our results, stress partially mediated the association between social isolation and depression symptoms (β = 0.33, SE = 0.01, *p* < 0.01). Further, resilience moderated the association between social isolation and depression symptoms (β = −0.04, SE = 0.02, *p* = 0.02) as well as the association between stress and depression symptoms (β = −0.06, SE = 0.01, *p* < 0.01). Detailed direct and indirect effects of predictors on the outcomes variable are presented in Table 4.

**Figure 2:**
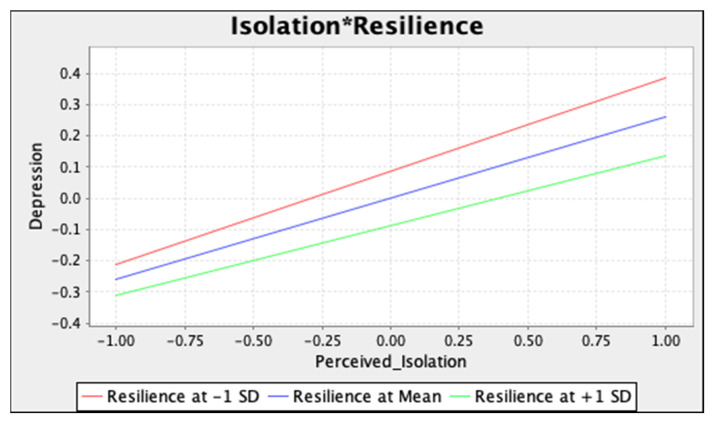
Simple slope test for the moderating effect of resilience on the isolation–depression association. Note. * represents the interaction term.

**Figure 3:**
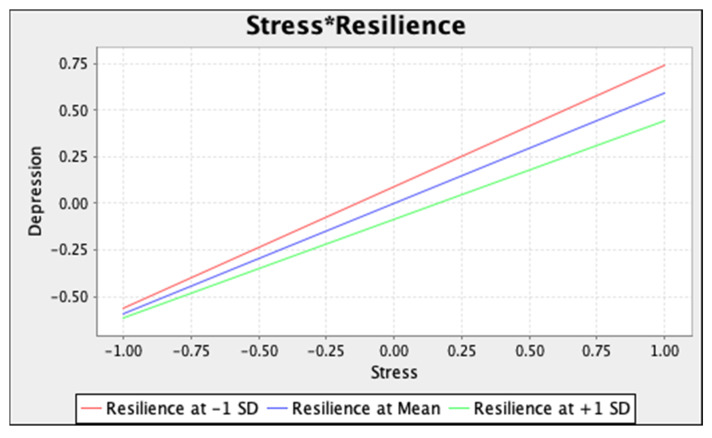
Simple slope test for the moderating effect of resilience on the stress–depression association. Note. * represents the interaction term.

To visually illustrate results from two moderation analyses, simple slope tests were conducted at three levels (i.e., 1 SD above mean, mean, and 1 SD below mean) of resilience (Figure 2 and Figure 3). 

## 4. Discussion

Restriction regulations during the pandemic may increase social isolation, leading to worsened mental health outcomes including depression symptoms. To provide empirical evidence that supports the development of mental health policies and interventions at universities, the current study examined the mediating role of stress and the moderating role of resilience in the isolation–depression association among university students. Based on our results, stress partially mediated the relationship between social isolation and depression symptoms. Further, we found resilience buffered the negative impact of social isolation and stress on depression symptoms. 

Consistent with previous studies, we found a positive association between social isolation and depression symptoms [40,41]. A high proportion of existing studies examined the association between social isolation and mental health symptoms in old adults. Only limited research has been conducted to determine such associations in young adults [42]. Indeed, it is generally believed that young adults may have larger social networks compared to older adults who are likely retired [43]. Yet, research has found that young adults (aged 18–29) reported higher levels of isolation feelings compared to their older counterparts (aged above 50), resulting in poor life satisfaction and psychological well-being [42]. Compared to middle-aged adults (i.e., aged 30–50), young adults tend to prefer quantity (i.e., number and time-spent of social interactions) over quality (i.e., perceived intimacy and satisfaction of interactions) in terms of social activities [44]. For university students (majority of whom are young adults), restriction measures may significantly limit the quantity of their social interactions, particularly face to face, leading to feelings of isolation and further contributing to depression symptoms. 

More importantly, we found that perceived stress mediates the association between social isolation and depression symptoms. Specifically, social isolation may lead to increased stress levels, which further contributes to depression symptoms. The mediating effect may be explained by differences in coping. It has been found that lonely individuals tend to use rumination as a coping strategy for negative situations [45]. Rumination has been identified as a major risk factor for depression symptoms and elevated stress [46,47]. An alternate coping mechanism that may prevent the worsening of depression symptoms is seeking support from social networks [18]. Further, it has been suggested that support from friends (compared to family and romantic partners) is perceived by young adults as helpful and reliable when in need [48]. During the pandemic, online interaction became an important way for university students to remain connected with their social networks, develop new friendships as well as receive social supports. A previous systematic review [49] suggested that active use of social media (e.g., engage in positive interactions, seek and receive social supports, and build social connections) was negatively related to depression and anxiety symptoms. Similarly, Facebook friends were found to increase perceived social support, reduce stress and resulted in greater well-being among university students [50]. During the pandemic, it is essential for higher education providers to encourage their students to stay socially connected despite being physically distanced from friends and peers. 

Our results demonstrate that resilience may buffer the negative impact of social isolation and stress on depression symptoms. Compared to students with low resilient levels, students high in resilience reported fewer depression symptoms during the pandemic despite the occurrence of social isolation and stress. As an ability to adapt to stressful situations, higher resilience level enables individuals to consciously appraise adverse conditions, reframe negative thoughts in a more useful manner, and utilize active strategies of coping with stressors (i.e., opposite to avoidant strategies such as behavioural withdrawal or substance use, [51]) when facing adversity [52]. As mentioned above, such an ability may serve as a protective factor against negative experiences associated with social isolation and stress, and in turn, reduce depression symptoms.

By highlighting the moderating role of resilience, our findings provide important implications for university administrators. Specifically, resilience is deemed as a trainable target for interventions [53,54,55]. Mindfulness-based interventions and physical exercise (i.e., High-Intensity Interval Training and Moderate-Intensity Training) have proved to be effective in improving resilience and reducing depression symptoms [55]. Demands for online interventions have been spiked during the pandemic. Compared to traditional face-to-face counselling services, online interventions offer advantages such as easy accessibility, scalability, and cost-effectiveness, and were preferred by young adults [56]. When facing the disruption of service delivery due to physical-distancing regulations and the potentially limited budgets for mental health services due to revenue shortfalls, it is of university administrators’ interest to leverage resources for online interventions. Based on our results, self-guided mindfulness training via smartphone apps as well as video-instructed exercise training may provide promising results in promoting resilience among students and should be considered as add-ons for current university mental health services.

Despite its input to existing literature, several limitations should be considered when interpreting the current findings. Firstly, there is a high proportion of female participants (i.e., 71.4%) in the current study, which may impact participants’ self-reported distress symptoms [57]. The overrepresentation of females in university students’ sample is well documented in psychological research [58,59]. The gender imbalance may cause concerns about the representativeness of the samples [58]. However, previous research has demonstrated that social isolation can predict depressive symptoms in both genders when tested separately [60]. Future research may benefit from using gender-balanced samples in order to control for potential confounds. Secondly, the current study did not assess participants’ previous existing mental health symptoms or diagnoses and did not include a measure or pre-pandemic mental health, which may be beneficial to include as a covariate in future studies in order to control for how these findings might have been impacted by the unique circumstances of the COVID-19 pandemic. Given the pandemic is still ongoing, future research is required to assess the longitudinal effect of social isolation on depression symptoms, and to assess this relationship post-pandemic. Thirdly, the current study adopted a cross-sectional design; thus, causation relationship between study variables may not be determined. Fourthly, data were collected from students enrolled in a single university in a country and state that implemented relatively severe COVID-19 physical-distancing restrictions for a prolonged period of time which may limit the generalizability of current findings amongst populations that did not experience such severe restriction measures. 

## 5. Conclusions

Social isolation (caused by university lockdown and social distancing measures) during the pandemic may contribute to university students’ depression symptoms both directly and through elevated stress levels. Students may need more internal/external resources to mitigate the negative consequences caused by the COVID-19 crisis and related measures. Resilience, a modifiable internal strength that may buffer the adverse effects of social isolation and stress on depression symptoms, should be targeted when developing prevention and early interventions. This may be especially important under physical-distancing measures when individuals have limited access to coping resources from external social networks. When dedicating resources during the pandemic, it will be important for university administrators to consider investing in building resilience among students. Online resources promoting awareness and mental health literacy, or online training for mindfulness and physical exercise that can be practised at home may be promising as preventions/early interventions to reduce depression symptoms among university students. Such interventions have advantages, including remote accessibility, cost-effectiveness and may serve as valuable add-ons to face-to-face counselling services that are considerably disrupted by restriction measures.

## Figures and Tables

**Table 1:** Descriptive statistics.

Variable	Mean	SD	Isolation	Stress	Resilience	Depression
Isolation	54.40	9.04	1.00			
Stress	22.10	7.25	0.55 **	1.00		
Resilience	18.77	4.80	−0.44 **	−0.58 **	1.00	
Depression	58.01	9.74	0.63 **	0.78 **	−0.56	1.00

Note. *n* = 1718; ** *p* < 0.01.

**Table 2:** Internal consistency/reliability and convergent validity.

Construct	Cronbach’s Alpha	rho_A	Composite Reliability	AVE
Depression	0.95	0.95	0.96	0.74
Social Isolation	0.89	0.89	0.92	0.75
Perceived Stress	0.89	0.90	0.91	0.51
Resilience	0.87	0.88	0.90	0.60

**Table 3:** Heterotrait–monotrait ratio (HTMT).

	Depression	Perceived Stress	Resilience
Perceived Stress	0.84		
Resilience	0.61	0.67	
Isolation	0.69	0.62	0.51

**Table 4:** Results of bootstrapping for direct/indirect effects examination.

	Beta	SE	T	*p*
Isolation > Depression	0.26	0.02	15.31	<0.01
Isolation > Stress > Depression	0.33	0.01	23.68	<0.01
Resilience > Depression	−0.09	0.02	4.80	<0.01
Isolation*Resilience > Depression	−0.04	0.02	2.38	0.02
Stress*Resilience > Depression	−0.06	0.01	4.34	<0.01

Note. * represents the interaction term.

## Data Availability

The data that support the findings of this study are available from the corresponding author upon request.
